# Fermented foods and cardiometabolic health: Definitions, current evidence, and future perspectives

**DOI:** 10.3389/fnut.2022.976020

**Published:** 2022-09-20

**Authors:** Katherine J. Li, Kathryn J. Burton-Pimentel, Guy Vergères, Edith J. M. Feskens, Elske M. Brouwer-Brolsma

**Affiliations:** ^1^Division of Human Nutrition and Health, Wageningen University & Research, Wageningen, Netherlands; ^2^Agroscope, Bern, Switzerland

**Keywords:** fermented foods, microorganisms, cardiometabolic disease, biomarkers, dietary assessment

## Abstract

Unhealthy diets contribute to the increasing burden of non-communicable diseases. Annually, over 11 million deaths worldwide are attributed to dietary risk factors, with the vast majority of deaths resulting from cardiometabolic diseases (CMDs) including cardiovascular disease (∼10 million) and type II diabetes (∼339,000). As such, defining diets and dietary patterns that mitigate CMD risk is of great public health importance. Recently, the consumption of fermented foods has emerged as an important dietary strategy for improving cardiometabolic health. Fermented foods have been present in the human diet for over 10,000 years, but knowledge on whether their consumption benefits human health, and the molecular and microbiological mechanisms underpinning their purported health benefits, is relatively nascent. This review provides an overview of the definitions of fermented foods, types and qualities of fermented foods consumed in Europe and globally, possible mechanisms between the consumption of fermented foods and cardiometabolic health, as well as the current state of the epidemiological evidence on fermented food intake and cardiometabolic health. Finally, we outline future perspectives and opportunities for improving the role of fermented foods in human diets.

## Introduction

### What is a fermented food?

For millennia, fermentation has been used as an effective method of food preservation and alcohol production (1). Fermentation of raw agricultural products can improve their nutritional qualities as well as impart new aromas and tastes. However, the increase in the popularity of fermented foods over the past decades has led to widespread misconceptions about what is required for a food to be considered “fermented.” Several definitions of fermented foods have been proposed over the years. One of the earlier documented descriptions, by Steinkraus (2), captures the biological complexity and transformative nature of food fermentation: “Fermented foods are food substrates that are invaded or overgrown by edible microorganisms whose enzymes, particularly amylases, proteases and lipases hydrolyze the polysaccharides, proteins and lipids to non-toxic products with flavors, aromas and textures pleasant and attractive to the human consumer.” However, this definition does not capture the intentional nature of food fermentation processes, and seemingly limits the transformative components in food to macronutrients. In 2021, the International Scientific Association for Probiotics and Prebiotics (ISAPP) provided a consensus statement on fermented foods, broadly defining fermented foods as: “Foods made through desired microbial growth and enzymatic conversions of food components” (3). Under this definition, fermented foods are those formed through a controlled process involving “desired” microorganisms. Importantly, the broad reference to conversions of “food components” suggests that the fermentation process could generate novel dietary compounds with distinct functional properties.

All fermented foods are procured *via* the actions of fermentative microorganisms. Microorganisms naturally present in the raw food matrix or the surrounding environment can initiate “spontaneous” fermentations, such as during the fermentation of cocoa beans using indigenous yeasts, lactic acid bacteria (LAB), and acetic acid bacteria (AAB) (4). However, large-scale industrial fermentations, which are commonplace, typically use starter cultures to ensure consistency in the end food product (5). Advances in food technology has also led to the application of alternative processing of foods that were traditionally fermented, resulting in non-fermented products, notably: pickled vegetables preserved in vinegar or brine, meat or fish preserved using salt, bread leavened using baking powder, and fresh cheeses curdled with vinegar or citric acid (Table 1).

**TABLE 1:** Classification of fermented foods adapted from Marco et al. (3).

Fermented
*Live microorganisms present* • Yogurt • Sour cream • Kefir • Most cheeses • Miso • Natto • Tempeh • Non-heated fermented vegetables • Non-heated salami, pepperoni and other fermented sausages • Boza, bushera and other fermented cereals • Most kombuchas • Some beers *Live microorganisms absent* • Bread • Heat-treated or pasteurized fermented vegetables, sausage, soy sauce, vinegar, and some kombuchas • Wine, most beers, and distilled spirits • Coffee and chocolate beans (after roasting) • Chocolate (after heat processing)

**Not fermented**

• Chemically leavened bread • Fresh sausage • Vegetables pickled in brine and/or vinegar • Chemically produced soy sauce • Salted or cured processed meats and fish

While the consumption of fermented foods tends to be synonymous with consuming a “dose” of live microorganisms, this is not always the case. The presence of live microorganisms in the ready-to-consume product is dependent on several factors: the phase of the fermentation (e.g., fermentation of cocoa beans in the food preparation phase versus fermentation of yogurt in the final product phase), whether the fermented food is heat-treated or if the microorganisms are intentionally removed (e.g., filtration of wine), as well as personal food preferences (e.g., cooked sauerkraut consumed in the Netherlands) (6). These factors create a key delineation (i.e., presence/absence of microorganisms) for the classification of different types of fermented foods with relevance to their health impacts.

### Fermented foods as a source of live microorganisms

In Western societies, the resurgent interest in the consumption of fermented foods can be credited to the explosion of research into the human microbiome (7, 8). Several studies have demonstrated that diet influences the structure and function of the gut microbiota (9, 10). It is believed that the consumption of fermented foods containing probiotics – “live microorganisms which, when administered in adequate amounts, confer a health benefit on the host” (11) – is an effective way to introduce potentially beneficial microorganisms to the intestinal tract and help manage a wide range of disorders associated with gut microbial dysbiosis. These include both intestinal disorders, such as behavior and (gut-)brain disorders, inflammatory bowel disease, irritable bowel syndrome, and coeliac disease, as well as extra-intestinal disorders, including allergy, asthma, obesity, metabolic syndrome, and cardiovascular disease (CVD) (12, 13).

The diversity of microorganisms found in fermented foods produced globally, as well as their functional properties, have been the subject of several comprehensive reviews (14, 15). Primarily, these include gram-positive (particularly LAB) and gram-negative bacteria, filamentous molds, and enzyme- and alcohol-producing yeasts (14). These microorganisms and their enzymes have varied functional roles, such as acting as antimicrobial agents (16), antioxidants (17), and fibrinolytic agents (15, 18). Recent advances in (meta)genomic sequencing are further expanding our understanding of the microbial diversity and functional potential of fermented foods, in particular bacterial and fungal species that have been less well-characterized due to difficulties in culturing these species (8, 19).

Another important aspect affecting the health impact of fermented foods is the amount of live microorganisms provided by the consumption of fermented foods. In a review by Rezac et al. (6), many fermented foods (cheese, yogurt, sausages, vegetables, cereals, sour beer, kombucha, fermented fish, and tempeh) were found to contain 10^5^–10^7^ colony forming units (CFU) of LAB/(mL or g), with cultured dairy products containing up to 10^9^ CFU/(mL or g). However, considerable variation was observed based on geographical region and sampling time, in addition to the manufacturing, processing, and storage conditions (6). Recently, Marco et al. (20) classified foods from the U.S. National Health and Nutrition Examination Survey (NHANES) 2001–2018 based on expert opinions on their levels of live microorganisms: low (< 10^4^ CFU/g), medium (10^4^–10^7^ CFU/g), and high (> 10^7^ CFU/g). They found that over 50% of children and adults were consumers of foods containing high levels of live microorganisms, and that the proportions of people consuming live microorganisms and per capita intake increased significantly over time (20). Although guidelines are lacking for the minimum dose of live microorganisms that should be consumed, the European Union (EU) health claim for yogurt and “improved lactose tolerance” stipulates that at least 10^8^ CFU of live starter microorganisms per gram of yogurt (21). Further, Derrien et al. (22) predicted that ingesting a dose of 10^10^ ingested bacterial cells would be sufficient to drastically shift the composition of the gut microbiota and impact the immune and neuroendocrine responses of the host.

### Fermented foods as a source of fermentation-derived metabolites

While not all fermented foods contain live microorganisms at the time of consumption, microbial activity during fermentation can still produce bioactive metabolites that could be beneficial to human health (23). The main fermentation processes can be grouped by the primary metabolites of fermenting microorganisms: alcohol and carbon dioxide produced by yeasts, acetic acid produced by AAB, lactic acid produced by LAB, ammonia and fatty acids produced by *Bacilli* and molds, and propionic acid produced by propionic acid bacteria (24). These metabolites parallel the end-products of the fermentation of undigested carbohydrate and protein by the gut microbiota, some of which (e.g., organic acids) have been positively associated with host gastrointestinal and immune health, lipid and protein metabolism, and appetite control (25).

Additionally, various secondary “bioactive” metabolites produced during fermentation are receiving increasing scientific interest for their functional properties. The types of metabolites produced depends on both the substrate and type of fermentation. For example, fermentation of milk results in the production of αs1- and β-casein peptide fragments from milk casein, which have been detected in several varieties of cheese (26). These bioactive peptides have angiotensin-converting enzyme (ACE) inhibitory activity and have also been reported to modulate opioid receptors in the gut epithelium (26). On the other hand, fermentation of multiple foods (e.g., cheese, sauerkraut) *via* a common fermentation pathway using LAB generates phenyllactic acids which help with food preservation as well as serve a physiological role of immune modulation (27, 28). Gamma-aminobutyric acid (GABA), which is enriched in sourdough bread through fermentation with LAB, has been shown to help control blood pressure and protect against CVD (29). Additionally, short-chain fatty acids (e.g., acetate, propionate, and butyrate) produced during microbial fermentation have shown hypocholesterolemic properties (30).

### Fermentation to enhance the nutritional quality of foods

In addition to introducing novel fermentation-derived metabolites and directly influencing nutrients, fermentation can also enhance the nutritional composition of the end food product or improve nutrient bioavailability. Various fermented foods have been shown to have enhanced nutritional attributes compared to their non-fermented counterparts (31–34). For example, it has been observed that levels of flavonoids, anthocyanins, and triterpenoids progressively increased during the fermentation of raw radishes, beets, and peppers (32). Fermentation of milk into cheese and yogurt has been shown to increase the levels of free amino acids detected in plasma, including α-amino butyric acid, alanine, asparagine, cysteine, glycine, glutamine, histidine, isoleucine, leucine, lysine, methionine, ornithine, phenylalanine, proline, serine, threonine, tryptophan, tyrosine, and valine (33, 34). Many micronutrients (calcium, phosphorus, A and B vitamins, potassium, zinc, and choline) also have a higher bioavailability in yogurt than in raw milk due to the acidity and fermentation process (35). Further, fermentation can also inactivate toxic dietary components and degrade anti-nutritional factors (36). Phytates present in cereals, legumes, and tubers are decreased during fermentation as a result of the activity of microbial phytases (36). The combination of fermentation and cooking also inactivates lectins from legumes that hinder nutrient absorption from the gastrointestinal tract (36).

## Fermented foods consumed in global and European diets

**FIGURE 1:**
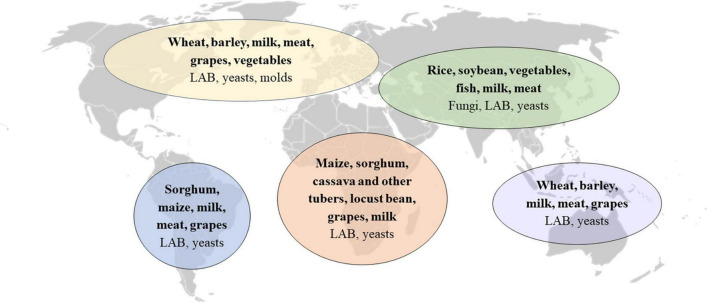
Predominant fermented food substrates and microorganisms in regions around the world. Adapted from Tamang et al. (40). LAB, lactic acid bacteria.

Over 5,000 types of fermented foods and beverages have been estimated to exist worldwide, contributing to 5–40% of the human diet (3, 37, 38). Every region and culture produces distinct fermented foods based largely on accessibility to different raw materials (Figure 1). The specific types and qualities of fermented foods consumed in different cultures around the world have been the subject of several comprehensive reviews (14, 39, 40). In the present review, we focus on fermented foods consumed in Europe.

In Europe, fermented foods were traditionally derived from fermentation of milk, wheat, barley, meat, grapes, and vegetables. Multiple varieties of cheeses, wine, beer, and dried/salted meats (including fermented meats) are designated as goods with “specific geographical origin” in the EU and have distinct historical and cultural significance (41). Several other fermented foods, such as coffee and chocolate, have origins in other cultures but have been normalized in European diets from ancient trade routes or colonization. It is expected that the varieties of fermented foods in European diets will continue to expand with globalization and consumer tastes for healthy and exotic flavors. A non-exhaustive overview of the types of fermented foods commonly consumed in modern European diets, as well as their raw materials and fermenting microorganisms, is provided in Table 2. It should be noted that although spirits (e.g., brandy, gin, whisky) are also technically considered to be fermented beverages, they were not further evaluated in this review, as their high alcohol content dominates the presence of other metabolites of fermentation that are interesting from a nutritional point of view.

**TABLE 2:** Substrates and microorganisms of predominant fermented foods consumed in European diets.

Fermented food	Substrate^a^	Fermenting microorganisms	Live microorganisms at time of consumption	References
**Cheese** (Afuega’l Pitu, Armada, Asiago, blue cheese, Brie, Burgos, Cabrales, Camembert, Cheddar, Comte, Danbo, Edam, Feta, Fontina, Galotyri, Gorgonzola, Gouda, Gubeen, Grana Padano, Havarti, Livarot, Limburger, Manchego, Monterey Jack, Mozzarella, Muenster, Parmesan, Puzzone di Moena, Pecorino Romano, Provolone, Stilton, Swiss, Swiss Gruyere, Tilsit)	Milk (bovine)	• **LAB:** *Lb. paracasei, Lb. rhamnosus, Lb. delbrueckii* subsp. *Bulgaricus, Lb. delbrueckii* subsp. *delbrueckii, Lb. delbrueckii* subsp. *lactis, Lb. helveticus, Lb. casei, Lb. plantarum, Lb. salivarius, Lc. lactis* subsp. *cremoris, Lc. lactis* subsp. *lactis, Leuc.* spp*., Ent.* spp. (*Ent. durans, Ent. faecium*), *Strep. thermophilus* • **Other gram-positive bacteria:** *Staph.* spp*., Brevibacterium linens, Propionibacterium freudenreichii* • **Fungi:** *Debaryomyces hansenii, Geotrichum candidum, P. camemberti, P. roqueforti*	Yes (most)	(6, 14)
**Yogurt**	Milk (bovine)	• **LAB:** *S. thermophilus, Lb. bulgaricus, Lb. acidophilus, Lb. casei, Lb. rhamnosus, Lb. delbrueckii* subsp. *bulgaricus, Lb. gasseri, Lb. johnsonii* • **Other gram-positive bacteria:** *B. lactis, B. bifidum*	Yes	(6, 14)
**Buttermilk**	Milk (bovine)	• **LAB:** *Lactococci, Lactobacilli*	Yes	(1, 6)
**Kefir**	Milk (bovine, ewe, goat, buffalo), kefir grains	• **LAB:** *Lb. paracasei* ssp. *paracasei, Lb. acidophilus, Lb. delbrueckii* ssp. *bulgaricus, Lb. plantarum, and Lb. kefiranofaciens, Lb. kefiri, Lb. brevis, Lb. casei* ssp. *pseudoplantarum, Lb. helveticus, Lb. lactis, Lb. lactis* ssp. *lactis, Enterococcus sp., Lc. Lacti, Lc. lactis ssp. cremoris, Leuc. mesenteroides* • **Other gram-positive bacteria:** *Bacillus* sp., *S. thermophilus* • **AAB:** *A. Aceti, A. rasens, A. fabarum, A. lovaniensis, A. orientalis, Gluconobacter frateurii* • **Yeasts:** *Sacc. cerevisiae, C. inconspicua, C. maris, Kluyveromyces marxianus, Kluyveromyces marxianus var. lactis, Dekkera anomala, Kazachstania kefir, Naumovozyma sp.*	Yes	(116)
**Bread** (white, wholegrain, sourdough)	Barley, rye, wheat	• **Yeasts:** *Sacc. cerevisiae, C. humili, Issatchenkia orientalis* • **LAB (sourdough only):** *Lb. sanfranciscensis, Lb. alimentarius, Lb. buchneri, Lb. casei, Lb. delbrueckii, Lb. fructivorans, Lb. plantarum, Lb. reuteri, Lb. johnsonii*	No	(14)
**Dried fermented sausage** (Salami, Salsiccia, Soppressata, Alheiras, Botillo, Chorizo, Salchicón, Pepperoni)	Pork or beef	• **LAB:** *Lb. plantarum, Lb. paraplantarum, Lb. brevis, Lb. rhamnosus, Lb. sakei, Lb. zeae, Lb. paracasei, Ent. faecalis, Ent. faecium, Leuc. mesenteroides, Ped. pentosaceus, Ped. acidilactici, W. cibaria, W. viridescens, Lb. sake, Lb. curvatus, Lb. plantarum* • **Other gram-positive bacteria:** *Micrococcus* spp., *Staph.* spp. • **Gram-negative bacteria:** *Enterobacteriaceae* • **Yeasts and molds**	Yes	(6)
**Sauerkraut**	Cabbage	• **LAB:** *Leuc. mesenteroides, Ped. pentosaceus; Lb. brevis, Lb. plantarum, Lb. sakei*	Yes	(6, 14)
**Fermented cucumbers**	Cucumbers	• **LAB:** *Leuc. mesenteroides, Ped. cerevisiae, Ped. acidilactici, Lb. plantarum, Lb. brevis*	Yes	(14)
**Fermented olives**	Olives	• **LAB:** *Leuc. mesenteroides, Ped. pentosaceus; Lb. plantarum Lb. pentosus/Lb. plantarum, Lb. paracollinoides, Lb. vaccinostercus, Lb. suebicus, Ped.* sp. • **Other gram-positive bacteria:** *Gordonia* sp. • **Gram-negative bacteria:** *Pseudomonas* sp., *Sphingomonas* sp., *Sphingobium* sp., *Sphingopyxis* sp., *Thalassomonas agarivorans*, • **Yeasts:** *C. cf. apicola, Pic.* sp., *Pic. Manshurica, Pic. galeiformis, Sacc. cerevisiae* • **Archaea:** *Halorubrum orientalis, Halosarcina pallid*	Yes	(14)
**Coffee**	Coffee cherries	• **LAB:** *Lc. Lactis, Leuc. Mesenteroides, Lb. plantarum, Lb. brevis* • **Other gram-positive bacteria:** *Bc. cereus, Bc. megaterium, Bc. subtilis, Bc. Macerans*	No	(117)
		• **Gram-negative bacteria:** *Serrati* sp*., Enterobacter agglomerans, Klebsiella pneumonia, Erwinia herbicola, Acinetobacter* sp*., Escherichia coli* • **Yeasts:** *Pic. anomala, Torulaspora delbrueckii, Rhodotorula mucilaginosa, C. ernobii, C. carpophila, Saccharomyces sp., Pic. caribbica, C. membranifaciens, Arxula sp., Hanseniaspora uvarum, Kluyveromyces sp., Kloeckera sp., C. xestobii*		
**Chocolate**	Cocoa pods	• **AAB:** *A. pasteurlanus, A. senegalensis* • **LAB:** *Lb. fermentum, Lb. ghanensis, Lb. brevis, Leuc. mesenteroides, Leuc. pseudomesenteroides, W. ghanensis, Lb. cacaonum, Lb. fabifermentans, W. fabaria, Fructobacillus pseudoficulneus, Lb. plantarum* • **Gram-negative bacteria:** *Enterobacteria, Tatumella ptyseos, Tatumella citrea* • **Yeasts:** *Sacc. cerevisiae, Kluyveromyces, Hanseniaspora uvarum, Hanseniaspora guilliermondii, Issatchenkia orientalis (C. krusei), Pic. membranifaciens*	No	(14, 118)
**Wine**	Grapes	• **Yeasts:** *Sacc. cerevisiae*, *C. colliculosa, C. stellata, Hanseniaspora uvarum, Kloeckera apiculata, Kl. thermotolerans, Torulaspora delbrueckii, Metschnikowia pulcherrima, Candida* sp. *and Cladosporium* sp.	No	(14)
**Beer**	Barley, hops	• **Yeasts:** *Sacc. cerevisiae, Sacc. carlsbergensis, Sacc. pastorianus*	No	(14)

A., Acetobacter; AAB, acetic acid bacteria; B., Bifidobacterium; Bc., Bacillus; C., Candida; LAB, lactic acid bacteria; Ent., Enterococcus; Lb, Lactobacillus; Lc, Lactococcus; Leuc. Leuconostoc; P., Penicillium; Ped., Pediococcus; Pic., Pichia; S, Streptococcus; Sacc., Saccharomyces; Staph., Staphylococcus; W., Weissella. ^a^Most common substrates listed, other substrates can also be used.

In a recent quantitative evaluation of food lists from food frequency questionnaires and 24-h recalls, it was determined that approximately 20% of the Dutch adult diet comprises fermented food items (42). These estimates are likely to reflect the prevalence of fermented food consumption in other European countries with similar diets. The fermented foods consumed primarily consisted of coffee, beer, wine, yeast-leavened bread products, chocolate, cheese, yogurt, quark, and buttermilk. Several less commonly consumed fermented foods were also captured by 24-h recalls, including certain fermented dairy products (sour cream, crème fraiche, yakult), sausages (salami and chorizo), fermented fish (salted herring, shrimp paste), vegetables (sauerkraut, fermented pickled vegetables), soy (miso, tempeh, soy sauce), and yeast-fermented desserts (doughnuts, pastries).

## The evidence between fermented foods and cardiometabolic health

### Proposed mechanisms

**FIGURE 2:**
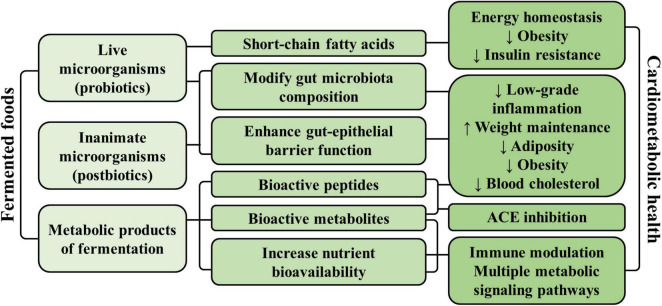
Summary of the main proposed mechanisms by which fermented foods may promote cardiometabolic health.

Several mechanisms have been proposed that support a role of fermented foods in promoting cardiometabolic health (Figure 2). As mentioned previously, fermented foods containing live microorganisms at the time of consumption may provide a source of probiotics, which can modulate both the composition and function of the host’s gut microbiota (22). Changes in gut microbial composition could enhance the integrity of the intestinal barrier and reduce low-grade inflammation associated with endotoxemia, which is speculated to be a mediator of obesity-related diseases (43). In animal models, consumption of dairy products with probiotics demonstrated greater cardiometabolic health benefits compared to consumption of dairy products without probiotics. In one such study, C57BL/6 mice on high-fat diets given kefir (a fermented dairy product) had reduced weight gain, hepatic steatosis, and low-density lipoprotein (LDL)-cholesterol levels compared to mice given milk (44). Mice given kefir also had higher levels of *Lactobacillus, Lactococcus*, total yeast, and *Candida* in the gut, which was strongly correlated with upregulated expression of fatty acid oxidation genes (*AOX*, *PPAR-α*) in both hepatic and adipose tissues. Reduced plasma levels of the pro-inflammatory cytokine IL-6 and down-regulation of the inflammation gene *MCP1* in adipose tissue was also observed. Evidence from several human trials also support a promising role for certain probiotic strains (primarily *Lactobacillus*) on weight maintenance, adiposity, obesity, and cholesterol levels, although further evidence is needed for their clinical relevance (45–49). Moreover, consumption of probiotics seems to modulate the function of the gut microbiota by increasing the production of short-chain fatty acids that impact energy homeostasis, obesity, and insulin resistance (50, 51). Given that there is a clear overlap between fermentation products and microbial activities in fermented foods and the gut microbiota, the literature on the health impact of the gut microbiota feeds back into the potential health benefits of fermented foods.

On the other hand, fermented foods that do not contain live microorganisms at the time of consumption can still be a source of postbiotics, defined as a “preparation of inanimate microorganisms and/or their components that confers a health benefit on the host” (52). The mechanisms of the non-viable microorganisms have been postulated to be similar to probiotics, such as helping to modulate the gut microbiota and enhance epithelial barrier function, as well as modulating host immune, metabolic, and signaling responses (52). In mouse models, consumption of heat-inactivated postbiotic preparation consisting of *Limosilactobacillus fermentum* and *Lactobacillus delbrueckii* resulted in altered gut microbiota composition and intestinal structure, indicating that the inactivated microorganisms maintain biological activity (53).

Importantly, both fermented foods containing live probiotics or inactive postbiotics contain end-products of fermentation (i.e., metabolites derived from or enhanced by the fermentation process), which could modulate multiple metabolic signaling pathways to improve overall cardiometabolic health. Many fermented foods are produced using LAB, which generates lactic acid, short-chain fatty acids, bioactive peptides, and polyamines with potential effects on cardiovascular, immune and metabolic health (52, 54). Fermenting bacteria can also influence cardiometabolic health by improving nutrient bioavailability, such as the bacterial production of vitamin K2 from vitamin K1, leading to a more potent activation of vitamin K-dependent proteins that affect multiple metabolic pathways (55). Other compounds may be present in fermented foods due to the presence of specific bacteria. For example, conjugated linoleic acid (CLA) that is associated with improved energy homeostasis (56) and which is already present in non-fermented dairy, may be elevated in fermented dairy foods due to the action of LAB or *Bifidobacteria* strains on linoleic acid (57).

### Epidemiological evidence for fermented foods and cardiometabolic health

#### Total (bacterial) fermented food intake

In the EPIC-NL cohort (*n* = 34,409) (58), total bacterial fermented food intake [which includes 78% dairy (yogurt, buttermilk and quark), 16% cheese, 4% meat (dried sausage), 2% vegetables (sauerkraut, pickles, olives), 0.1% soya (tempeh), and 0.2% vinegar] was not significantly associated with both all-cause mortality [hazard ratio (HR) 1.00, 95% CI 0.88–1.13], and CVD-related mortality (HR 1.04, 95% CI 0.83–1.30). Subgroup analyses did show a moderate inverse association between cheese and CVD mortality, particularly from stroke (HR 0.59, 95% CI 0.38–0.92). In this study, fermented foods made using yeast as the main starter culture (bread, wine, beer, alcoholic drinks) or by endogenous enzymes/microorganisms (cocoa, coffee, tea) were intentionally excluded. Aside from this report, no studies have investigated the impact of total fermented food consumption on CMDs and associated risk factors.

#### Fermented foods containing live microorganisms

In Europe, fermented dairy products comprise the largest group of fermented foods consumed that contain live microorganisms. Thus, numerous studies have examined the cardio-protective effects of fermented dairy foods, both in the context of and separately to total dairy intake. For instance, the Prospective Urban Rural Epidemiology (PURE) study (*n* = 136,384, 21 countries, five continents) (59) showed that higher intake of total dairy foods (> 2 servings/day versus no consumption) was associated with significantly lower risks of total CVD (HR 0.84, 95% CI 0.75–0.94), cardiovascular mortality (HR 95% CI 0.77, 0.58–1.01), major CVD events (HR 0.78, 95% CI 0.67–0.90), and stroke (HR 0.66, 95% CI 0.53–0.82), which seemed to be particularly driven by intakes of yogurt (HR total CVD 0.86, 95% CI 0.75–0.99) and milk (HR total CVD 0.90, 95% CI 0.82–0.99). In contrast, the Northern Sweden Health and Disease Study (*n* = 108,065) showed that higher intakes of milk were associated with an increased risk of myocardial infarction (HR 1.17, 95% CI 1.03–1.34) and type II diabetes (HR 1.23, 95% CI 1.10–1.37) in men, but not women (60). Intakes of fermented milk, butter and cheese were not significantly associated with these CVD-related outcomes. Stratified analysis by fat content suggested that lower fat dairy products were associated with increased risk of the CVD-related outcomes assessed. The authors hypothesized that this may be attributed to other factors (i.e., compensation of calories from dairy with that of other food groups, consumption of other foods in the diet in addition to low-fat dairy, or to possible cardio-protective effects of dairy fats). Effects of fermented dairy intake on individual CMD risk factors have been examined as well, including blood lipids (61–65), hypertension (66–70), body mass index and obesity (71–76), type II diabetes, glycemia and insulin homeostasis (60, 74, 77, 78), also with conflicting findings. A review of 16 meta-analyses by Gille et al. (79) showed weakly beneficial albeit inconsistent links between fermented dairy products and several CMD risk factors (Table 3). The strongest evidence in the review was observed between yogurt on risk factors of type II diabetes (79).

**TABLE 3:** Summary of the systematic reviews and meta-analyses between dairy foods and various cardiometabolic disease risk parameters^a^ [adapted from Gille et al. (79)].

	Total dairy	Milk	Cheese	Yogurt
**Prospective studies**				
CVD	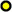	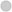	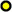	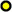
CAD/CHD	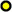	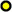	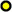	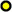
Stroke	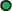	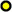	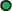	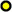
Hypertension	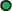	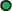	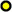	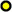
Metabolic syndrome	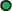	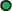	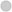	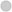
Type II diabetes	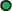	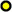	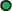	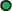
**Intervention studies**				
LDL-cholesterol	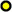	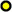	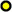	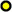
HDL-cholesterol	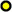	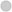	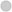	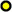
Fasting triglycerides	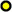	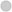	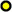	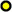
Postprandial triglycerides	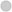	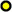	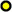	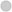
LDL size	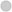	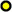	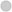	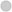
Apolipoprotein B	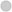	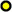	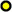	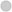
Non-HDL cholesterol	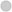	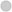	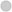	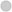
Cholesterol ratios	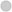	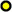	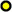	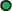
Inflammation	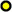	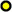	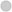	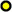
Insulin resistance	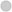	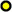	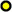	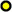
Blood pressure	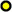	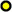	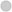	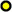
Vascular function	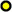	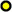	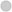	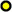

CAD, coronary arterial disease; CHD, coronary heart disease; CVD, cardivascular disease; HDL, high-density lipoprotein; LDL, low-density lipoprotein.

^a^Colors represent the overall findings of the studies: favorable (green), neutral or no effect (yellow), uncertain or undetermined (gray). No adverse associations were observed.

#### Fermented foods containing inactivated microorganisms

Several other fermented foods whereby live microorganisms have been inactivated or removed (coffee, wine, beer, and chocolate) have also been investigated for their effects on CMD risk. Due to the abundance of literature on these foods, we focused on summarizing the results of systematic reviews and meta-analyses. In terms of coffee, previous prospective studies and meta-analyses did not show associations between coffee consumption and coronary heart disease (CHD) risk (80, 81). However, a recently updated systematic review and meta-analysis of prospective cohort studies observed an inverse non-linear association between moderate habitual coffee consumption and CVD risk (82) with the lowest risk reported at 3 to 5 cups of coffee per day [relative risk (RR) 0.85, 95% CI 0.8–0.9]. Here, genetic polymorphisms may be an important consideration to further interpret the heterogeneous impacts of coffee consumption on CVD-related outcomes (83).

Several meta-analyses have shown that moderate intake of red wine (e.g., 270 mL/day) may be protective effect against CHD, while extremely high intakes have deleterious effects (84–86). Using data from the EPIC Spanish cohort (*n* = 15,630 men and *n* = 25,808 women), Arriola et al. (84) found that “moderate” (5–30 g of alcohol/day) and “high” (30–90 g alcohol/day) intakes of wine by men reduced CHD risk compared to low or no intakes (HR 0.40; 95% CI 0.25–0.64 for moderate intakes; HR 0.44; 95% CI 0.28–0.69 for high intakes). Similarly, a systematic review of prospective studies indicated that moderate beer consumption of up to 16 g alcohol/day (equivalent to 1 drink/day) for women and 28 g/day (1–2 drinks/day) for men may also have a protective effect against CVD and mortality compared to non-alcohol or occasional drinkers (87). However, a recent meta-analysis of controlled clinical trials of beer intake found that beer drinkers had elevated total cholesterol, high-density lipoprotein (HDL)-cholesterol and apolipoprotein A1, and flow mediated dilation compared to non-beer drinkers, but no differences in LDL-cholesterol, triglycerides, blood pressure, or other biochemical markers of inflammation (88). Overall, the current state of the evidence on the impact of wine and beer intake on different CMD risk parameters remain equivocal.

For chocolate, multiple meta-analyses have found that consumption of chocolate was inversely associated with CHD, stroke, and type II diabetes; however, effective categories or levels of intake differed among the studies and need to be further clarified, and several aspects of study quality needs to be improved (89–92).

Aside from these abovementioned fermented foods, to our knowledge, no well-designed prospective studies have examined the cardiometabolic health impacts of other fermented food products consumed in European (or global) diets.

### Limitations of the current evidence

While a plethora of studies have examined associations between the intake of certain fermented foods (fermented dairy, coffee, wine, beer, and cocoa) and cardiometabolic health outcomes, closer examination of the methods of these studies reveals limitations and inaccuracies which could obscure the interpretations of the results. In many studies, non-fermented foods have been misclassified as fermented food products. For example, in an association study between fermented dairy intake, diet quality, and cardiometabolic profile, Mena-Sánchez et al. (93) defined fermented dairy products as “low-fat yogurt, whole-fat yogurt, and all types of cheese (petit Swiss; ricotta; fresh cheese; cottage; and semi-cured and cured cheeses such as Cheddar, Manchego, and Emmental).” Similarly, Kostinen et al. (94) included cottage cheese under total fermented dairy products when examining associations between fermented and non-fermented dairy products and risk of coronary heart disease. Ricotta, cottage cheese, and fresh cheeses are produced by curdling of milk with acid, and are thus generally not considered to be fermented (95). In light of the recently clarified definitions of fermented foods, a more critical classification scheme is thus required to correctly classify fermented foods prior to examining associations between their intake and health outcomes. Furthermore, almost all studies to date have relied on self-report measures to assess the intake of fermented foods (58, 93, 94). The subjective nature of these tools can result in inaccurate estimates of the levels of fermented food intake. The characteristics and limitations of self-report dietary assessment tools are described in detail below, as well as an opportunity to improve the accuracy of assessing fermented food intake through more objective measures.

## Opportunities and future perspectives in research for fermented foods

### Improving the dietary assessment of fermented foods

One of the criticisms of population-based studies in nutritional epidemiology is the inability to accurately capture the foods consumed in the diet and their levels of intake, contributing to inconsistent evidence between fermented foods and health, and weakening their potential translation to clinical and public health applications (96, 97). Common dietary assessment methods such as 24-h recalls (i.e., a detailed account of all foods consumed in the previous 24 h self-reported by participants or inventoried by trained interviewers) and food frequency questionnaires (FFQ) (self-reported intakes of a pre-determined list of foods, typically in the past month to year), have advantages and drawbacks (98). They rely on (subjective) self-reporting, and their precision depends upon the devotion, diligence, and memory of the participants. Such errors can lead to reduced power from non-systematic reporting errors, systematic reporting errors, as well as underestimated or overestimated findings in association studies.

To circumvent the limitations of self-report dietary measurement tools, researchers have looked to food intake biomarkers (FIBs) as objective measures of dietary intake (99). The identification of new FIBs has been driven by the application of metabolomics in nutritional research, which allows for a comprehensive measurement of all low molecular weight molecules in biological samples (100). Identifying a single FIB for a specific food can be a challenging task, since most dietary compounds commonly occur in many different foods. Different food sources may also contain common FIBs from a shared food processing method or metabolic pathway. This is particularly relevant to fermented foods, where common microbial fermentation pathways (e.g., lactic fermentations) can result in the production of similar sets of metabolites. At the same time, the diverse raw material substrates used for fermentation can be a source of unique parent compounds. In these cases, multi-marker approaches, consisting of a combination of non-specific yet complementary biomarkers, could better inform the intake of fermented foods (101). Multi-metabolite panels have been suggested for wine, cocoa, and bread (101), but remain to be exploited for other fermented foods. Thus, there is value in identifying FIBs for fermented foods that could improve the dietary assessment of these foods for future studies, inform on their nutritional quality, and elucidate the mechanisms of action that underpin the health benefits of fermented foods. In addition, there is a need to validate candidate FIBs for fermented foods that have emerged from non-targeted and targeted nutritional metabolomics studies in real-life, non-controlled situations.

### Expanding the discovery of food intake biomarkers for globally consumed fermented foods

There is a bias in the literature for investigating a narrow list of fermented foods. In a recent systematic review of FIBs of fermented foods, it was shown that the vast majority of studies were conducted for coffee, wine, cocoa, beer, and bread, while only a smattering of other types of fermented foods were represented (102). For some foods, such as fermented soy, there appears to be ample research but the studies primarily target a list of food group-level biomarkers (e.g., isoflavones found in all soy products), whereas FIBs specifically associated with fermented soy intake were not investigated. While these are common foods consumed in Western/European diets, due to globalization, many of nutritious and delightful fermented food products that are indigenous to other parts of the world are now available locally. Thus, the current literature on FIBs of fermented foods presents an “incomplete” picture of the diversity of fermented foods consumed in modern globalized diets. To address this, further discovery-driven studies on the identification of FIBs for less common fermented foods and condiments in Europe (sourdough, sauerkraut, salami, Worcestershire sauce) and globally (kombucha, kefir, tempeh, kimchi, soy sauce) are warranted. Further, quantification of the most promising FIBs could help in order to use these biomarkers to calibrate self-reported fermented food intakes to clarify diet-disease associations (103). Expanding such research could eventually have an impact on public health, such as through refining dietary guidelines for fermented foods.

### Development of a fermented food-specific food frequency questionnaires

While self-report dietary assessment tools are often perceived to be inaccurate due to response bias and measurement error, their qualities of being non-intrusive and non-resource intensive still supports their use in large population-based studies. In addition, well-designed FFQs can produce valid and reproducible estimates to rank participants according to their levels of intake (42). Since most FFQs are not designed to capture fermented foods but rather common foods in the diet of a specific population, there is conceivable value in developing a FFQ specific for fermented foods, which could better capture the habitual intake of fermented foods.

In order for a fermented food-specific FFQ to be effective in accurately capturing the types and levels of fermented foods, researchers must first agree on what foods are considered to be “fermented.” Just recently in 2021, the International Scientific Association for Probiotics and Prebiotics (ISAPP) convened an expert panel to create a common definition for fermented foods (3), which is a step toward unifying future research on the compositional and health qualities of fermented foods. Additionally, consumer misconceptions about fermented foods could be simultaneously addressed. A survey conducted among ∼200 university students revealed that nearly two-thirds were unfamiliar with the term “fermented dairy products,” and a similar percentage were unsure whether several cultured dairy products were fermented (104). Such unfamiliarity with fermented foods would yield inaccurate estimates of the types and levels of fermented foods consumed in the diet. To circumvent this, improvements in food legislation could make it easier for consumers to identify fermented foods in the marketplace based on clear food labeling (3). Such food labeling should clearly indicate a food or certain food brands as being “fermented,” and provide additional nutritional information, such as the presence or absence of live microorganisms, as well as compositional aspects of the fermented food. This information could also be linked to a comprehensive, food composition database to help facilitate future research.

### Better understanding the composition of different fermented foods and their documentation in food databases

Fermented food products are complex and multi-faceted. The composition of fermented foods reflects untransformed compounds from the raw food substrate, transformed raw material compounds by the fermentation process, and novel metabolites produced by fermentative microorganisms. Additionally, the presence of live microorganisms (or even inactivated microorganisms which can still be bioactive) presents a unique additional compositional layer (3, 52). Metabolomics and metagenomics analyses coupled with bioinformatics and machine learning could better capture the molecular composition of fermented foods. Concurrently, documenting the composition of different fermented foods and their meta-data in food composition databases (new or existing) is a critical step. Food composition databases are widely used in nutrition and health research to provide information on the nutritional content of foods. However, current food composition databases only report a limited number of nutrients, which only represent a minute fraction of the thousands of distinct chemicals in our food (105). Additionally, in the modern food supply there exists a diverse range of manufactured fermented food products. The same generic fermented food can have many unique formulations depending on the manufacturing source. Here, the use of branded food databases (where information on food composition is provided by manufacturers, food monitoring, and crowdsourcing) are gaining momentum and could be a valuable resource in comprehensively capturing the ingredients within branded fermented foods (106–108). Documenting the composition of fermented foods could open new research avenues for understanding how their consumption affects health and disease. Additionally, such information could also benefit food industries to formulate products with improved nutritional qualities.

### Understanding the contribution of fermented foods to healthy and sustainable diets: fermentation as “Agriculture 2.0”

Finally, food fermentation could be thought of as a versatile tool to help extend the world’s food supply. Global food and agriculture production accounts for a quarter of the world’s greenhouse gas emissions, and the global population is poised to reach 10 billion by 2050 (109, 110). With so many mouths to feed, there is a pressing need to reflect on how to improve the sustainability of human diets to best protect the natural environment. A recent report from the EAT-Lancet Commission advised consumers to eat more plant-based foods and less animal-based foods in order to relieve the human footprint on food systems (111). Fermented foods could play an important role in achieving healthy and sustainable plant-based diets. For millennia, food fermentation has been used as a strategy to avoid food waste by increasing the shelf life of fresh foods. In the modern food supply chain, fermentation of plant-based foods can be an effective strategy to improve their shelf life, storage, and transport (112). The consumption of many protein-rich vegetable or legume alternatives to meat are constrained by the high presence of anti-nutrients, which can be reduced by food fermentation. In addition, fermentation can have an effect on increasing the palatability of plant-based foods by introducing new flavors and reducing unpleasant flavors. On the other hand, fermentation may be used as a strategy to reduce meat consumption (rather than replacing it completely), through the development of novel fermented food products with mixed protein and animal sources (113).

Additionally, fermented foods have an important role in promoting a circular economy, by utilizing waste products from the food industry (e.g., peels, seeds, pulp) and generating new, value-added foods (114). While it is known that fermentation of foods can also increase their nutrient density (thus reducing the need to consume large amounts of foods), industrial fermentations also require a lot of energy (115). Thus, more work is required to elucidate the nutritional and sustainable trade-offs of fermented food production and consumption. Overall, if fermented foods can be consumed as part of a sustainable dietary pattern, they can help improve the environmental impact of food production, promote human health (reducing the risk of CMDs), and in turn, promote economic prosperity.

## Conclusion

Fermented foods have been consumed by humans for over 10,000 years, and their renewed popularity in modern diets emphasizes the need to fully elucidate their effects on cardiometabolic health. Although some studies have suggested that the consumption of fermented foods could benefit cardiometabolic health, the evidence is still unclear. This could be partly due to the misclassification of fermented foods, and the use of subjective dietary assessment tools to estimate their intake, in large epidemiological studies. Several future research directions could help disentangle the relationships between fermented foods and cardiometabolic health: improving the dietary assessment of fermented foods (through identification of FIBs and development of a fermented food FFQ), better understanding the composition of different fermented food products, and exploring the role of fermented foods as part of healthy, sustainable diets.

## Author contributions

KL wrote the original draft of the review and prepared the figures and tables. EB-B, KB-P, GV, and EF critically reviewed the manuscript. All authors contributed to the study concept, and read and approved the final manuscript.
